# Making It PEACHY: Creation of an Innovative Immersive Simulation Day Promoting Empathetic, Attentive Communication for Holistic Care for Year 4 Medical Students

**DOI:** 10.1192/bjo.2024.313

**Published:** 2024-08-01

**Authors:** Vatsala Mishra, Archanaa Elankovan, Ian Winston

**Affiliations:** Barts Health NHS Trust, London, United Kingdom

## Abstract

**Aims:**

This team of simulation fellows and a final-year medical student at a London teaching hospital created an innovative simulation course for fourth-year medical students with the aim to supplement existing undergraduate psychiatry teaching by providing additional opportunity to practice clinical skills. The course allowed students to practice and improve advanced communication skills across a range of inpatient and community settings across GP, A&E, medical and psychiatric environments, with themes exploring psychiatry, heightened emotional states and biopsychosocial influences on mental and physical health in a safe, ethical manner, supplementing the teaching provided on clinical placements.

**Methods:**

The course was fully mapped to the university curriculum as well as the Health Education England Future Doctor Vision and the Medical Licensing Assessment content map. Scenarios were written by trained simulation faculty in conjunction with specialty experts across all core specialties for fourth year medical students including obstetrics and gynaecology, paediatrics, psychiatry, and healthcare of the elderly. Scenarios were created to reflect local demographics with addition of detailed social history and population health information. This involved creation of simulated patients from multicultural backgrounds, with limited English or other communication needs, and representation of numerous gender expressions, sexual orientations, and a range of mental health and neurodevelopmental needs.

**Results:**

The pilot course took place on May 2nd with 7 student participants following approval from senior education stakeholders. During debriefs, participants differentiated between psychiatric symptoms and non-pathological human experiences, and reflected on how and why the patient in front of them is presenting the way that they are, with regards to social determinants of physical and mental health. They were also guided to reflect upon the technical and non-technical learning objectives of each scenario including use of Crisis Resource Management principles. Quantitative and qualitative feedback was collected through use of Likert-scales and white space questions; feedback showed heightened confidence and competence in core skills including psychiatric history taking, mental state examination and risk assessment, as well as core communication skills such as explaining a new diagnosis and managing heightened emotion.

**Conclusion:**

Feedback shows the pilot successfully met its aims and enhanced undergraduate training, filling an educational need. Next steps would include formally approaching the university to discuss implementation of the course into the core curriculum. Additional refinements would include further consultation with service users and people with lived experience and consideration around use of actors to ensure complex subjects such as immigration and neurodivergence are portrayed ethically and accurately.

